# When Can Species Abundance Data Reveal Non-neutrality?

**DOI:** 10.1371/journal.pcbi.1004134

**Published:** 2015-03-20

**Authors:** Omar Al Hammal, David Alonso, Rampal S. Etienne, Stephen J. Cornell

**Affiliations:** 1 School of Biology, University of Leeds, Leeds, United Kingdom; 2 Center for Advanced Studies (CEAB-CSIC), Blanes, Spain; 3 Groningen Institute for Evolutionary Life Sciences, University of Groningen, Groningen, Netherlands; 4 Institute of Integrative Biology, University of Liverpool, Liverpool, United Kingdom (current address); University of Chicago, UNITED STATES

## Abstract

Species abundance distributions (SAD) are probably ecology’s most well-known empirical pattern, and over the last decades many models have been proposed to explain their shape. There is no consensus over which model is correct, because the degree to which different processes can be discerned from SAD patterns has not yet been rigorously quantified. We present a power calculation to quantify our ability to detect deviations from neutrality using species abundance data. We study non-neutral stochastic community models, and show that the presence of non-neutral processes is detectable if sample size is large enough and/or the amplitude of the effect is strong enough. Our framework can be used for any candidate community model that can be simulated on a computer, and determines both the sampling effort required to distinguish between alternative processes, and a range for the strength of non-neutral processes in communities whose patterns are statistically consistent with neutral theory. We find that even data sets of the scale of the 50 Ha forest plot on Barro Colorado Island, Panama, are unlikely to be large enough to detect deviations from neutrality caused by competitive interactions alone, though the presence of multiple non-neutral processes with contrasting effects on abundance distributions may be detectable.

## Author Summary

In order to predict and mitigate the response of ecological communities to global change, we need to understand the processes that allow multiple species to coexist in close proximity. A classic idea in Ecology is that species coexist because they occupy different “niches”. However, random processes such as dispersal could also explain species coocurrence, without invoking niche differentiation. “Neutral” models embody this idea, omitting niche differentiation and assuming all species are identical. Such models are mostly statistically consistent with the relative abundances of tree species in tropical forests, but statistical procedures always contain an element of uncertainty and many other models could also be consistent with a particular data set. We compute how strong the non-neutral processes would need to be in order for their effect to be detectable in data sets of different sizes. We find that the largest ecological data sets currently available, such as the 50 hectare plot on Barro Colorado Island in Panama, are not large enough to distinguish between neutral and non-neutral models, unless multiple non-neutral processes are at work. This means that other types of pattern need to be studied, or larger data sets collected, in order to understand the mechanisms behind forest biodiversity.

## Introduction

The extent to which ecological processes can be inferred from macroecological patterns has long been debated [1–4]. The appearance of common patterns in species abundance distributions (SADs) for different communities suggests that the same ecological mechanisms structure these communities [5, 6]. However, it is now thought that many patterns describing communities are rather insensitive to these processes [7–11]. For example, empirical SADs are in many cases found to be statistically consistent with Hubbell’s neutral theory [12–17], but this does not mean that communities are truly neutral because non-neutral models can predict similar [8–10, 18, 19] or even identical [4] patterns. This raises the question of whether anything can be inferred from fitting it to SAD data [3, 4, 20].

Neutral theory has been criticized for the biological processes it omits, but non-neutral models that give qualitatively or even exactly the same predictions can be equally artificial and unrealistic [4, 18, 21]. Neutral theory has many virtues [22–24] and in many ways it is more complete in scope than competing niche theories [25]. It describes community dynamics at the individual level, treating births, deaths, and dispersal as stochastic processes. It is susceptible to rigorous statistical tests, because unlike many other demographic models the likelihood of obtaining a particular community or sample can be computed exactly [26–30]. Even the controversial neutral assumption that interactions between individuals do not depend on species identity is inspired by biological reality; Hubbell observed that, in tropical forests, all species compete for light—and, therefore, space [31]. This means that neutral theory should be a good starting approximation for communities of sessile species that compete for a common resource, such as space (e.g. tropical trees or coral reefs). More realistic models will include non-neutral processes, such as interactions that depend on species identity [32, 33], but neutral theory can act as a null model for assessing the weight of evidence for such processes.

Although the SAD may be rather insensitive to the introduction of non-neutrality, this does not mean that it is identical for any kind of non-neutral effects [34]. While there may be patterns or scales for which some processes are undetectable, e.g. due to central limit theorem-like effect [19, 35, 36], strong interactions between individuals can structure communities and it is in some cases possible to detect their existence from inspection of the SADs [3, 33, 34]. If a data set is found to be consistent with neutral theory, we should therefore be able to infer that some particular non-neutral processes are not present in that community, or at least are not strong enough to produce detectable deviations from neutrality in a data set of this size.

In this paper, we present a power calculation for neutral theory. Our purpose is to estimate an upper bound for the strength of non-neutral processes in tropical forest data sets [37–39] that have been found to be consistent with neutral theory [13]. To do this, we fit the standard neutral model (SNM) to data sets generated by a non-neutral model, and compute the probability of rejecting neutral theory. We test the neutral null hypothesis using a maximum likelihood approach (using an exact expression [26] for the likelihood of a sample from the SNM), where *p*-values are evaluated by a parametric bootstrap procedure.

The power of a statistical test is defined as the probability that the null hypothesis is rejected when it is indeed false. The power therefore depends on which alternative hypothesis is true. In this paper, we focus on two classes of non-neutral processes: interspecific competition, and intrinsic (density independent) fitness differences between species. Interspecific competition is one of the classic mechanisms that promote coexistence [33, 40, 41], whereas differences in fitness represent the fact that the mean environmental conditions in a particular area of habitat will tend to favour one species over another. These represent opposite ends of a spectrum of possible non-neutral models, because symmetric interspecific competition tends to lead to equal abundances among species, whereas intrinsic fitness differences tend to lead to highly uneven abundances. While these are only two examples out of an infinite set of non-neutral models, our method provides a blueprint for computing the detectability of any type of non-neutral process.

Full details of our models are given in the Methods section. Our models are similar in structure to Hubbell’s standard neutral model (SNM), in that we consider stochastic population dynamics in a local community where strong density dependence regulates the total community size to *J* individuals, coupled by immigration to a much larger metacommunity. We consider two models of interspecific competition: one, which we shall denote HL, is a multi-species stochastic Lotka-Volterra model similar to that studied by Haegemann and Loreau [33]; the other, denoted by PC, has stochastic Ricker-like dynamics as studied by Pigolotti and Cencini [42]. Our model of intrinsic differences in fitness, denoted by IF, assumes that the fecundity of each species is a randomly generated variable. Each of our models has a single parameter that determines how strong the non-neutral processes are. In model HL, parameter *γ* represents the relative difference in strength between interspecific and intraspecific interactions, so that when *γ* = 0 the dynamics are neutral whereas when *γ* > 0 coexistence is promoted. In model PC, parameter *c* determines the difference between inter- and intra-specific density dependence, so *c* = 0 corresponds to neutral interactions and non-neutrality becomes stronger as *c* increases. In model IF, the fitness of each species is generated from a Gamma distribution with shape factor 1/*k*, so that when *k* = 0 all fitnesses of the species are the same and the local dynamics are neutral.

As in the SNM, local diversity is maintained by a fraction *m* of all recruits being immigrants from a metacommunity with fixed relative species abundances. The proportion of immigrants of different species follow their relative abundance in the metacommunity. We consider two cases: in case LOGS the metacommunity is described by a logseries with fundamental diversity constant *θ*, and in case EVEN the metacommunity has *S*_*T*_ species which all have equal abundance. A logseries distribution can arise from many processes, including but not restricted to neutral dynamics [43]. We considered the even metacommunity because it represents a metacommunity limit of our local community dynamics, and as a result represents a contrasting, extremely non-neutral, limit to the logseries. When coupled to the LOGS metacommunity, each of our models should be equivalent to the SNM when the local dynamics are neutral (when *γ, c*, or *k* equals zero). As the dynamics are made more non-neutral, the deviations from the SNM should become stronger, and we expect the power of the test of the neutral null model to increase. However, when coupled to the EVEN metacommunity, the models are not equivalent to the SNM even when the local dynamics are neutral. In this case the power of the test could be high even if the local dynamics were neutral, though if *J* is very small the statistical power could still be low.

Our study consists of two parts. First, we explore how the parameters of our models affect the probability of detecting non-neutral processes. For a real community we do not know *a priori* the appropriate parameters to use, so we need to choose the parameters so that the alternative model gives comparable patterns to the empirical data. In the second part of our study, we estimate the power of tests of neutrality for empirical data from three New and Old World tropical forests, including Barro Colorado Island (BCI) in Panama. Our power calculation provides an estimate of the smallest sample size that is needed to detect non-neutrality of known intensity, and of the range strengths of non-neutrality needed to reject neutrality for a given species abundance data set.

## Results

### Power calculation for fixed non-neutral model parameters

**Fig 1:**
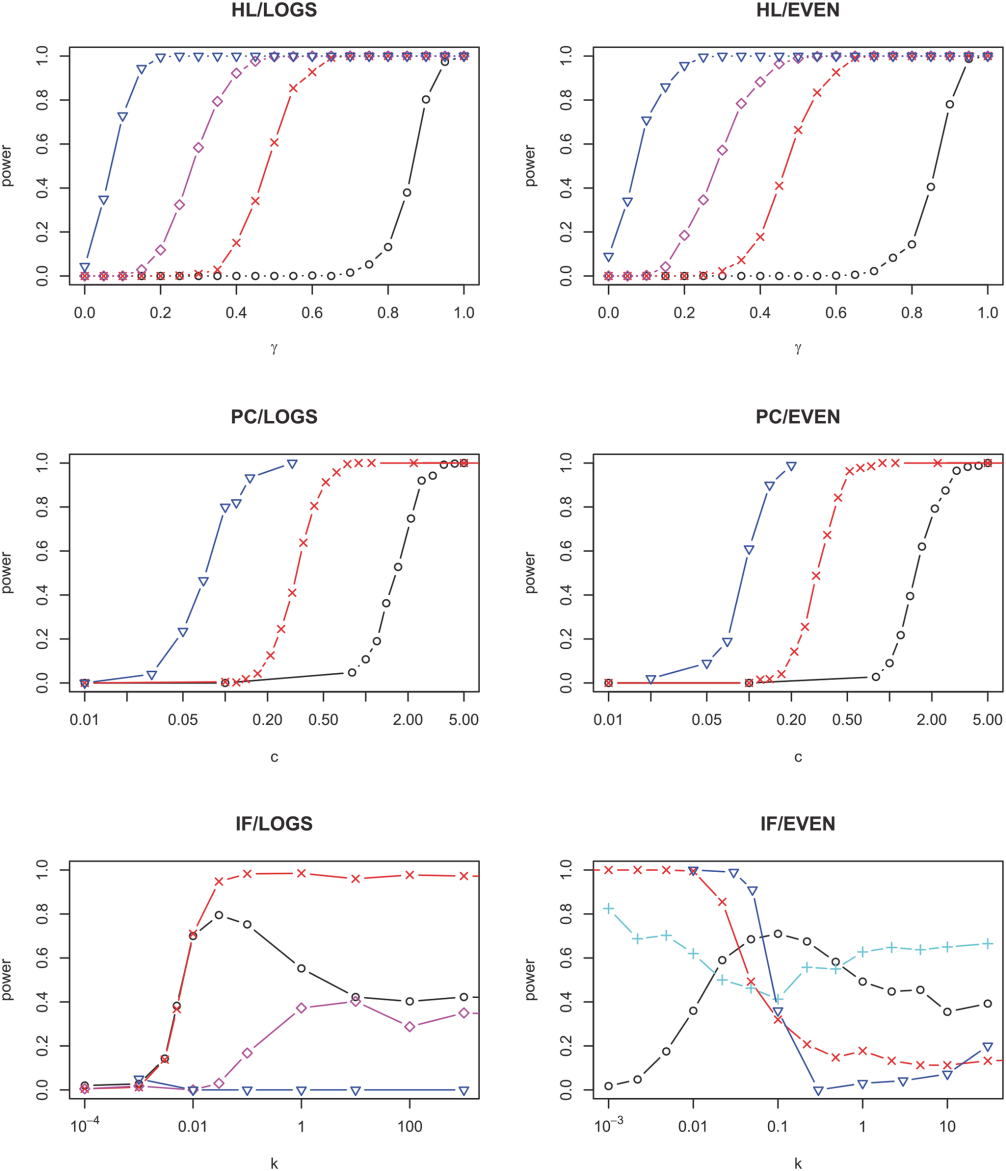
Probability of rejecting the neutral null hypothesis as a function of the strength of non-neutral processes. Top row: HL model; middle row: PC model; bottom row: IF model. The local dynamics are strictly neutral in the limits to *γ* = 0, *c* = 0, or *k* = 0 respectively. The panels on the left assume a logseries metacommunity with biodiversity parameter *θ*; panels on the right assume a community with *S*_*T*_ species with equal abundances. Different colours/symbols correspond to different local comunity sizes, as follows: Black circles *J* = 200; red multiplication signs *J* = 2000; cyan plus signs *J* = 1000; magenta diamonds *J* = 5000; blue triangles *J* = 20000. The other paramerers are: HL/LOGS: *θ* = 50, *m* = 10^−4^; HL/EVEN: *S*_*T*_ = 2000, *m* = 10^−4^; PC/LOGS: *θ* = 50, *m* = 10^−4^; PC/EVEN: *S*_*T*_ = 200; IF/LOGS: *θ* = 100, *m* = 0.1; IF/EVEN: *S*_*T*_ = 100, *m* = 0.1.

The strength of non-neutral processes affects the sample size that is required in order to have a good chance of rejecting the neutral hypothesis (see Fig. 1). When interspecific interactions are non-neutral (models HL and PC, top and middle row of Fig. 1), we see a simple pattern: as the strength of non-neutral processes is increased (*γ* or *c* increases from zero), the power of the test increases. In addition, for these models the power of the test increases as the local community size *J* is increased. This shows that any strength of non-neutrality, however weak, can in principle be detected provided the data set is large enough. However, the system sizes needed to give a significant power may be too large to be empirically accessible when local dynamics are nearly neutral (*γ* or *c* close to zero). For the LOGS metacommunity, and when the local dynamics are strictly neutral (*γ* = 0 for model HL or *c* = 0 for model PC), the models are equivalent to the SNM, and the power is equal to the threshold *p*-value for statistical significance (0.05 in our study). However, for the EVEN metacommunity the power can be higher than this threshold even when the local dynamics are neutral, because the immigration process makes the model no longer equivalent to the SNM (though this is not visible for the relatively small values of *m* used with models HL and PC in Fig. 1)

However, the patterns are rather more complicated for model IF (bottom row of Fig. 1). The power is again low when the local dynamics are neutral (*k* → 0) and the metacommunity follows a logseries (bottom left panel). However, the power does not increase monotonically as the non-neutrality parameter *k* is increased. This is because strong selection rapidly leads to dominance by a single species [44], especially in small communities, which is a pattern that can also arise from the SNM if the immigration parameter *m* → 0. Moreover, the power no longer increases monotonically when the local community size *J* increases; for example the power for *J* = 2000 in Fig. 1, bottom left, is higher than for *J* = 200, 5000, or 20000. This appears counterintuitive because statistical power should increase monotonically with sample size. However, *J* represents more than just the amount of data available: it is a parameter which interacts non-linearly with the model dynamics. In the IF model, for instance, it determines whether the dynamics are in the strong or weak selection limit, and it also plays a nonlinear role in the SNM.

**Fig 2:**
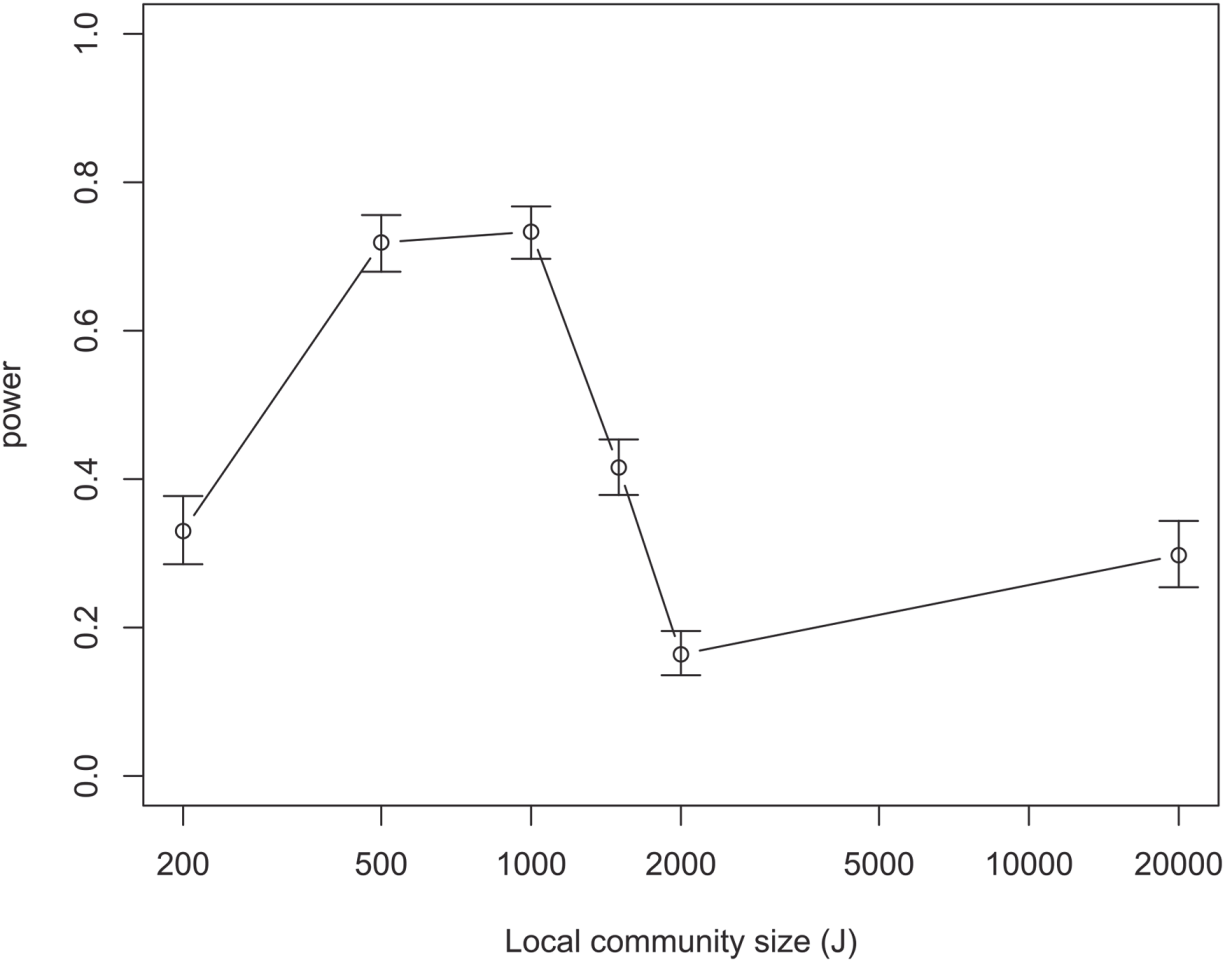
Probability of rejecting the neutral null hypothesis as a function of local community size *J* for model IF/EVEN in the limit *k* → ∞. Here, *m* = 0.1 and *S*_*T*_ = 100. Error bars are 95% Jeffreys confidence intervals.

To illustrate this effect, we can consider the limit *k* → ∞ of model IF/EVEN. In this special case, there is a single dominant species, relative to which all other species have zero fitness. All local recruits will therefore be of the dominant species, though other species will also be present due to immigration. This case is particularly simple because the species identity of each individual in the local community is the dominant species with probability 1−*m*(1–1/*S*_*T*_), and each of the other species with probability *m*/*S*_*T*_. We find that the power of the test of neutrality is low at small *J*, increases to a maximum at an intermediate value of *J*, and then decreases as *J* increases again (Fig. 2). Because, for the non-neutral model in this limit, *J* is nothing more than a sample size—a community of size 2*J* can be constructed by adding two communities of size *J*—this non-monotonic relationship between power and *J* must be due to the nonlinear role played by *J* in the community dynamics in the neutral null model.

The two other model parameters (immigration rate and diversity of the metacommunity) also strongly affect the chance of rejecting the neutral hypothesis. The parameter *m* describes the probability that a newly born individual in the local community is an immigrant from the metacommunity, so the local community resembles the metacommunity more closely as *m* is increased; when *m* = 1, the local community is effectively a random sample from the metacommunity and local dynamics are irrelevant. Increasing *θ* in model LOGS, and increasing *S*_*T*_ in model EVEN, lead to more diverse metacommunities, and as a result tend to increase diversity in the local community.

**Fig 3:**
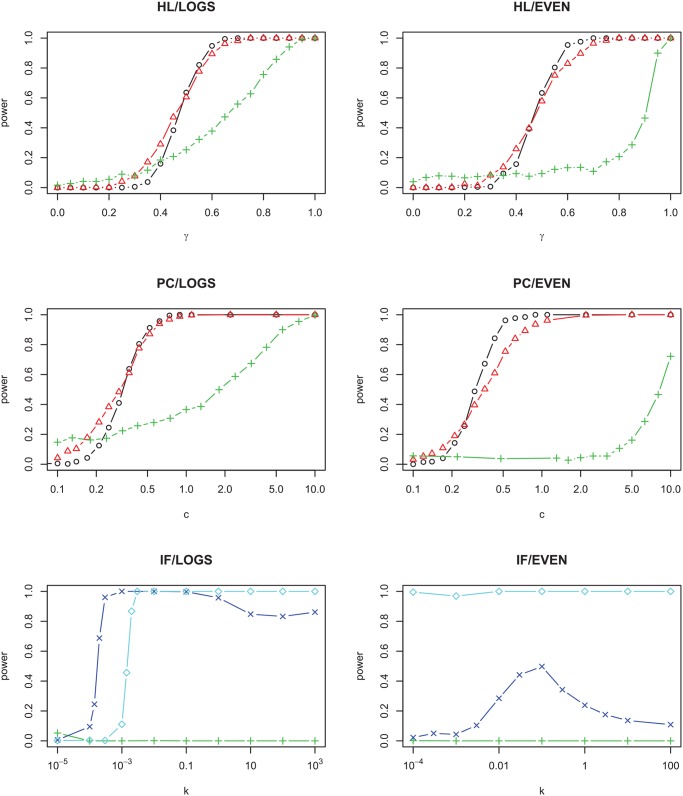
Power of rejecting the neutral null model as a function of the strength of non-neutrality, for different rates of immigration from the metacommunity. Back circles: *m* = 10^−4^, red triangles: *m* = 10^−3^; green plus signs *m* = 0.01; blue multiplication signs *m* = 0.03; cyan diamonds: *m* = 0.1. For HL/LOGS and PC/LOGS *θ* = 50; for IF/LOGS *θ* = 1000; for HL/EVEN, PC/EVEN and IF/EVEN *S*_*T*_ = 200. In all cases *J* = 2000.

**Fig 4:**
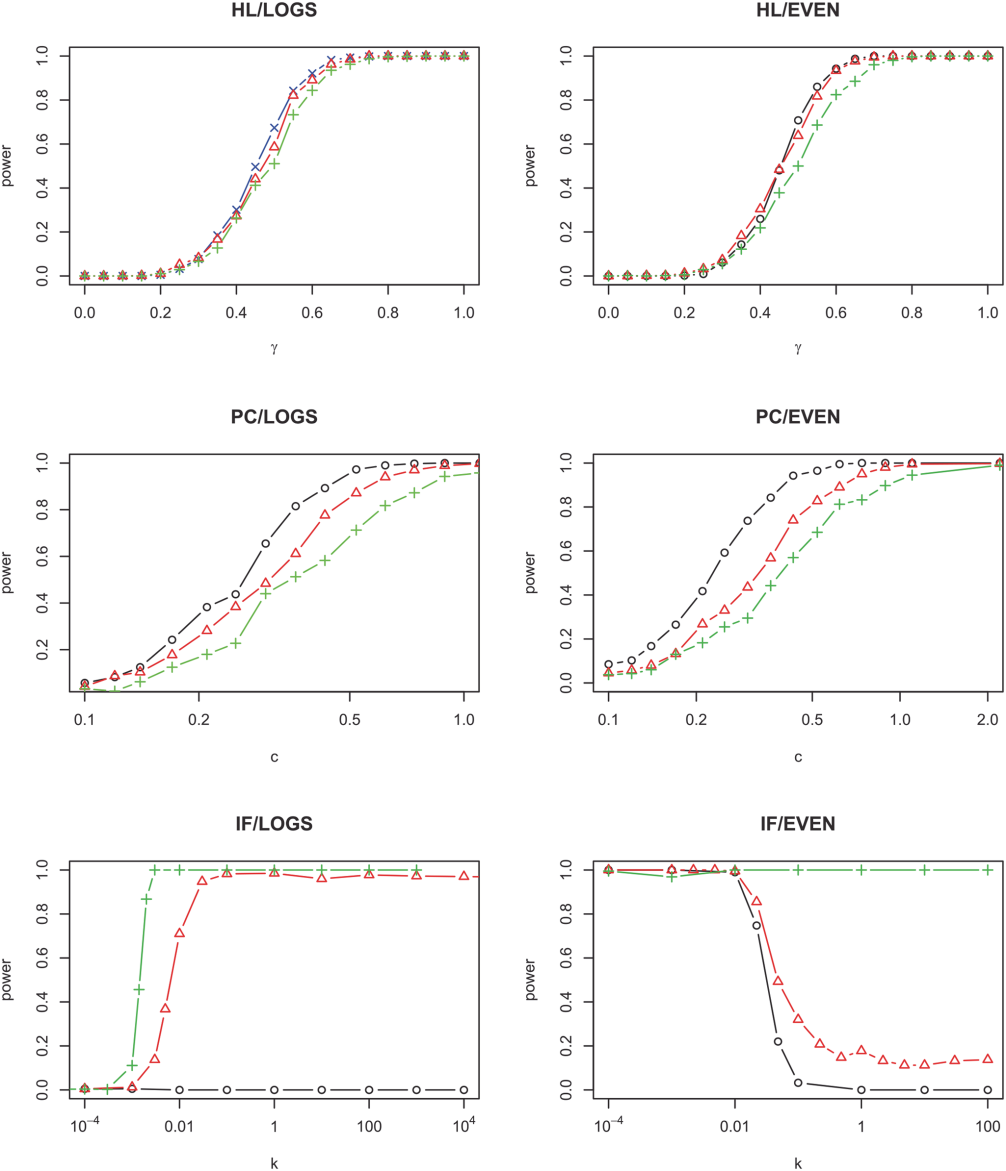
Probabilty of rejecting the neutral null model as a function of the strength of non-neutrality, for different levels of diversity in the metacommunity. The parameters are: for HL/LOGS: *m* = 10^−3^ and *θ* = 25 (blue multiplication signs), *θ* = 50 (red triangles), *θ* = 200 (green plus signs); for PC/LOGS, *m* = 10^−3^ and *θ* = 10 (black circles), *θ* = 50 (red triangles), *θ* = 200 (green plus signs); for HL/EVEN and PC/EVEN, *m* = 10^−3^ and *S*_*T*_ = 20 (black circles), *S*_*T*_ = 50 (red triangles), *S*_*T*_ = 500 (green plus signs); for IF/LOGS, *m* = 0.1 and *θ* = 10 (black circles), *θ* = 100 (red triangles), *θ* = 1000 (green plus signs); for IF/EVEN *m* = 0.1 and *S*_*T*_ = 50 (black circles), *S*_*T*_ = 100 (red triangles), *S*_*T*_ = 200 (green plus signs). In all cases *J* = 2000.

For models HL and PC, either increasing *m* (top and middle rows Fig. 3), or increasing the diversity of the metacommunity (either increasing *θ* or *S*_*T*_ as appropriate, top and middle rows Fig. 4), reduces the power of the test. It is clear why increasing *m* should reduce the power for models HL/LOGS and PC/LOGS, because the local community is then more like a logseries, and hence more like the SNM. The power changes very little between *m* = 10^−4^ and *m* = 10^−3^, reflecting the much greater importance of local dynamics on the patterns when *m* is small. It is less clear why increasing *θ* should reduce the power of the test, though it is worth noting that both increasing *θ* and increasing *m* have the effect of increasing the local richness. Increasing *m* or *S*_*T*_ in models HL/EVEN and PC/EVEN also increases the local richness, and while it is not obvious why this should make the model resemble the SNM, we find that it also reduces the power of the test.

For model IF, the effect of both *m* and the metacommunity diversity on the statistical power can be non-monotonic (bottom row, Figs. 3 and 4). For model IF/LOGS when *k* ≈ 10^−3^, for instance, for *θ* = 1000 the power for *m* = 0.03 is higher than for *m* = 0.1 or 0.01 (bottom left, Fig. 3); for *m* = 10^−3^ the power for *θ* = 100 is higher than for *θ* = 10 or 1000. On the whole, however, both *m* and the community diversity tend to increase the power of the test, which is the opposite effect from what is seen in models HL and PC. This is because the local dynamics tend to lead to monodominant states, which as explained before are indistinguishable from the SNM even if their origin is highly non-neutral. Processes which increase the local diversity allow the non-neutral features of this model to be better detectable.

It is interesting to note that the shape of the relationship between power and *c* for model PC in Fig. 1, middle row seems to be independent of *J*. This would be a very useful relationship if it were found to hold in general, because power calculations for large *J* are very expensive computationally and this would enable us to estimate power by varying *c* as a proxy for *J*. We find, however, that this behaviour is not preserved for other parameter values. We did not find any simple way to summarise the dependence on model parameters evident in Figs. 1, 3, and 4 that would enable us to estimate the power outside of the parameter range we tested explicitly.

### Power calculation for large forest surveys

Our aim is to explore the detectability of non-neutrality in data sets of different sizes. SNM has been found to be statistically consistent with several large tropical forest data sets [12–17], but this does not mean that SNM is an exact description so a power calculation gives us, in principle, an upper bound for the degree of non-neutral processes in these systems. We do not know *a priori* the appropriate non-neutral parameter values for these forests, but we can choose model parameters so that the model data match a number of features of the empirical data. Specifically, we chose model parameters so that the community size, mean species richness, and mean Shannon diversity of sample data sets from the non-neutral model are the same as in the empirical data set. More details are given in the Methods section.

We estimated the power of neutrality for empirical data similar to three data sets collated by the Centre for Tropical Forest Science (CTFS, data available online at http://www.ctfs.si.edu). The determination of appropriate model parameters, and the power calculation itself, is very computationally expensive, so we performed this for three candidate strengths of non-neutrality, and for models PC and IF only. The results are summarised in tables 1–4.

**Table 1:** Fitted parameters for CTFS data sets and probability of rejecting the neutral null hypothesis, for model PC/LOGS.

Forest	*c*	(*θ, m*)	Power (Jeffreys 95% confidence interval)
BCI	0.1	(40.04, 0.2398)	0.00 (0.00–0.03)
	1	(38.44, 0.3256)	0.02 (0.00–0.04)
	10	(36.15, 0.600)	0.00 (0.00–0.05)
Pasoh	0.1	(183.2, 0.04686)	0.00 (0.00–0.03)
	1	(168.1, 0.06710)	0.01 (0.00–0.03)
	10	(138.5, 0.1708)	0.00 (0.00–0.03)
Lambir	0.1	(219.4, 0.2641)	0.01 (0.00–0.05)
	1	(217.3, 0.2515)	0.01 (0.00–0.03)
	10	(205.0, 0.4031)	0.01 (0.00-0.05)

**Table 2:** Fitted parameters for CTFS data sets and probability of rejecting the neutral null hypothesis, for model PC/EVEN.

Forest	*c*	(*S*_*T*_, *m*)	Power (Jeffreys 95% confidence interval)
BCI	0.01731	(∞, 0.0008181)	0.10 (0.05–0.17)
Pasoh	0.1	(1154, 0.00353)	0.06 (0.03–0.10)
	0.7523	(∞, 0.001876)	0.20 (0.13–0.29)
Lambir	0.1	(4506, 0.003329)	0.02 (0.00–0.05)
	0.2858	(∞, 0.002771)	0.01 (0.00–0.05)

The cases where *S*_*T*_ = ∞ correspond to the largest values of *c* for which the model could reproduce the three characteristics of the empirical data.

**Table 3:** Fitted parameters for CTFS data sets and probability of rejecting the neutral null hypothesis, for model IF/LOGS.

Forest	*k*	(*θ, m*)	Power (Jeffreys 95% confidence interval)
BCI	10^−4^	(40.70, 0.1888)	0.00 (0.00–0.03)
	10^−4^	(57.50, 0.02441)	0.02 (0.00–0.06)
Lambir	10^−4^	(221.0, 0.2394)	0.01 (0.00–0.05)
	10^−4^	(361.5, 0.02922)	0.01 (0.00–0.05)

For these forests with *k* = 10^−4^, there were two distinct parameter sets that match the characteristics of the empirical data. No solutions were found for *k* ≥ 0.01. Pasoh is not included because no parameter sets consistent with the empirical data were found with *k* ≥ 10^−4^.

**Table 4:** Fitted parameters for CTFS data sets and probability of rejecting the neutral null hypothesis, for model IF/EVEN.

Forest	*k*	(*S*_*T*_, *m*)	Power (Jeffreys 95% confidence interval)
BCI	10^−4^	(232, 0.0166)	1 (0.975–1)
	0.01	(232, 0.134)	1 (0.975–1)
	1	(232, 0.517)	1 (0.975–1)
Pasoh	10^−4^	(675, 0.0232)	1 (0.976–1)
	0.01	(672, 0.181)	1 (0.976–1)
	1	(672, 0.648)	1 (0.976–1)
Lambir	10^−4^	(1018, 0.0236)	1 (0.981–1)
	0.01	(1006, 0.186)	1 (0.976–1)
	1	(1006, 0.650)	1 (0.975–1)

For model PC/LOGS, we find that the power of the test of neutrality is extremely low when the model parameters are chosen to match three statistics of the empirical data, whatever the strength of the non-neutrality (Table 1). This is because the non-neutral process tends to increase the evenness in the community, so when *c* is increased the fitted immigration rate needs to be increased in order to match the Shannon index in the empirical data. In other words, the parameters of this model need to be close to neutral (either *c* small, or *m* close to 1) in order to agree with the empirical data, so the power of the test is low.

By contrast, for model IF/EVEN the power of the test is very high for model parameters that reproduce the characteristics of the empirical data (Table 4), even for the smallest strength of non-neutral processes we considered. This is because both the metacommunity and the local dynamics are non-neutral, but the local dynamics tends to lead to very uneven (i.e. monodominant) communities while the metacommunity tends to increase the evenness of the community. These processes need to be in balance in order for the model to match the diversity and richness of real data, and as a result the fitted model is far from neutral.

For model PC/EVEN, both the local dynamics and the metacommunity tend to lead to even abundance distributions, and as a result it was only possible to find parameters so the model matches the empirical data when the strength of the non-neutrality was sufficiently weak (Table 2). The largest value of *c* for which the model is consistent with the empirical forest data was found to be when *S*_*T*_ → ∞, in which limit the immigration process behaves effectively as a speciation process. It was found that when the model was fitted to the Pasoh data with *c* = 0.7523, the probability of rejecting the neutral null hypothesis was 0.20. The power of the test was much lower when *c* = 0.1, and was always low when the model was fitted to the Lambir data. The model could not reproduce the richness and Shannon diversity of the BCI data set unless *c* < 0.1.

For related reasons, the probability of rejecting the neutral null model was low for model IF/LOGS when fitted to the empirical data. Here, both the non-neutral local dynamics and the metacommunity tended to lead to highly uneven abundance distributions, and as a result the Shannon index produced by the model was very low unless *k* was small. It was not possible to fit the model to Pasoh when *k* ≥ 10^−4^, or to BCI or Lambir when *k* ≥ 0.01. While there were two discrete parameter sets each that matched the richness and evenness of Pasoh and Lambir (one where *θ* was low and *m* high, and one where *θ* was higher and *m* lower), the power of the test with these parameters was always low.

## Discussion

Our power calculation shows that, in principle, non-neutrality would be detectable in large enough SAD data sets or when non-neutral processes are strong enough (see Fig. 1). This contradicts the suggestion that the SAD for large samples will approach the same canonical form, and that larger sampling efforts would consequently be futile [35]. Indeed, the SNM has been rejected using SAD data from very large phytoplankton communities [45], and we found that the SNM could also be rejected for the tropical tree species abundances of Yasuni National Park (see Methods). Our results also show that independent niches can be distinguishable from neutrality, contrary to suggestions by Chisholm and Pacala [36], because the test is most powerful when the species in our model HL undergo independent stochastic logistic dynamics (i.e. when *γ* approaches 1, see Fig. 1). Our HL model with *γ* = 1 differs from the independent-niche models of Chisholm and Pacala [36] and Haegeman and Etienne [19] by having strong intra-specific density dependence, so the marginal distributions are very different from the SNM.

However, we conclude that tropical forest abundance data sets, on the scale collected by CFTS, might not be large enough to detect even strongly non-neutral interspecific interactions. As shown in Tables 1 and 2, statistical power remains very low as *c* (the parameter measuring the intensity of inter- versus intra-specific competition) is varied. This is because the model parameters required to give the same richness and evenness in the empirical data are themselves close to neutral, either because *c* is small or because the metacommunity follows a logseries distribution and *m* is close to 1 (in which case the local community strongly resembles a neutral-like metacommunity). This result is in agreement with the good fits of some species-independent neutral models to a large number of SADs for very diverse communities [46]. It is interesting to note that Volkov et al. [47] estimated that interspecific species interactions are many times smaller than intraspecific interactions in tropical forests, but we cannot apply their results to our models because they did not include immigration from a metacommnunity.

On the other hand, we did find that a combination of non-neutral processes producing opposing effects on the local community led to high statistical power for parameters consistent with empirical CTFS data. In model IF/EVEN, intrinsic local fitness differences tend to decrease richness and evenness, whereas the even local community increases richness and evenness. This means that both processes can be strong while still producing levels of richness and abundance consistent with the empirical data. Du et al. [10] noted that non-neutral processes which have opposing effects on relative abundance distributions can lead to abundance distributions that resemble neutral theory, but our investigation shows that they can still be distinguished from SNM in some cases. We can therefore conclude that such a combination of strongly non-neutral processes is *not* present in data sets for which the SNM is not rejected, such as the three CTFS forests we studied in this paper.

The power of a statistical test generally depends on three factors: first, the sample size; second, statistical significance as measured by the threshold *p*-value used to assess significance; and third, the effect size, which quantifies departures from the null hypothesis. In our analysis, effect size is encoded in the parameter values of the non-neutral model under consideration (respectively, *γ, c*, and *k* for models HL, PC, and IF). When density dependence is non-neutral (models HL and PC), power increases as interactions become more non-neutral (see Fig. 1). However, for non-neutral intrinsic fitness (model IF) the power of the test depends non-monotonically on *k* (Fig. 1, bottom row). These patterns can be understood from the effect that these parameters have on diversity patterns—strong non-neutrality (*k* large) in model IF leads to monodominance, which is indistinguishable from the neutral model with strong dispersal limitation (*m* very small).

Our results highlight the fact that the parameter *J* plays a more complicated role for these models than the sample size in standard power calculations, because the power does not always increase monotonically with *J* (Fig. 2). In most standard statistical tests, a “sample” consists of a number of statistically independent measurements. In an ecological community (or a model thereof), the individuals are not statistically independent because of their interactions (whether within or between species). This is true even in the SNM: an equilibrium community of size *J* can be generated as a hypergeometric subsample of a community with larger *J* [48], but the individuals are not independent because this represents sampling without replacement. This means that the community size *J* plays a nonlinear role and is not a straight analogue of the sample size in standard statistical tests, so statistical power does not necessarily increase monotonically with *J*.

The dependence on other parameters can also be non-monotonic; for example, for metacommunity model LOGS the local community will resemble a log series (and therefore be indistinguishable from the SNM) in the limit *m* → 1, but the power does not decrease monotonically with *m* for model IF/LOGS (see Fig. 3). Increased metacommunity diversity decreases statistical power for models HL and PC (Fig. 4), which echoes the observation that higher local diversity leads to SADs that look more like those created by the SNM even in the presence of niche structuring [19, 36]. This suggests that it might be easier to quantify non-neutral interactions in less diverse forests [19]. However, this is not true for all types of non-neutral processes: for model IF the power increases when the metacommunity diversity is increased.

Unfortunately, we were unable to find any general rules to allow us to extrapolate the power calculation outside of the parameter range we simulated. The power calculations in this paper are very computationally expensive, and it would be unfeasible for us to repeat them for *J* much larger than ∼ 30000 individuals. Moreover, to do this we would need to know how parameters *γ* (for model HL) and *c* (for model PC) are affected by *J*. Our notation tacitly assumes that each species is sensitive to mean population densities over the whole community, but in real systems, where individuals of a species are clumped together, an individual will only interact with nearby individuals so the values of *γ* and *c* might depend on *J*. We are therefore unable to estimate the factor by which CTFS data sets would have to be enlarged for us to distinguish model PC from the SNM.

In this paper, we have analysed a range of non-neutral scenarios: non-neutral density dependence affecting mortality or recruitment; non-neutral differences in intrinsic fitness; neutral-like or extremely non-neutral metacommunity. These processes have contrasting effects on the SAD, so arguably represent the extremes of a spectrum of possibilities. Nevertheless, there are many types of community processes which are not encompassed by our models. For example, trophic or mutualistic interactions are not present in our models and should lead to very different patterns of abundance, though these are more likely to be relevant in other systems than the tropical forests on which we focus. Similarly, dynamics that lead to multimodal SADs should be relatively easy to distinguish from neutrality [34, 49]. We have made a number of simplifying assumptions to keep the number of parameters manageable, but our framework could still be used to perform a power calculation for any type of non-neutrality that can be incorporated in a simulation model. It would also be preferable to perform power calculations for spatially explicit models, which represent a more realistic dispersal process and can readily be simulated [50]; however, for our test we would need a likelihood for the spatially explicit neutral model, which is not currently available.

Our results have refined as well as quantified in a statistical sense the suggestion [3, 36, 51, 52] (but see also ref. [53]) that SAD data do not have sufficient resolving power to assess the importance of non-neutral processes in structuring forest communities. Our study shows that even large-scale tropical forest data sets are not large enough, or are too diverse, to detect non-neutral species interactions using the SAD alone. However, we would not expect to see a good fit to the SNM if these forests contained multiple processes with opposing effects on richness and evenness. Patterns that include more information, such as multiple samples [27], spatiotemporal changes [54, 55] or phylogenetic data [56, 57], are likely to be much more revealing about the processes that generated them. Provided it is possible to compute the likelihood for obtaining such patterns in a neutral model, our approach can be adopted to calculate the sampling effort needed to detect and quantify non-neutral processes, and understand the forces that structure communities.

## Methods

This section describes (i) our non-neutral alternative models, and methods for generating samples from them; (ii) our method for testing whether to reject the neutral null hypothesis for a particular data set; (iii) the method for combining (i) and (ii) to give a power calculation; (iv) the method for estimating model parameter values in order to estimate the statistical power of particular experiments.

Our non-neutral models are similar in structure to the standard neutral model (SNM), i.e. Hubbell’s metacommunity-local community model [26, 31, 48], but adapted to include non-neutral processes. As with the SNM, strong local density dependence is assumed to keep the local community size fixed at *J* individuals. A fraction *m* of all recruits immigrate from a “metacommunity”, which is assumed to be large enough for the relative abundances (*P*_*i*_ for the *i*’th species) to be effectively static in time. This immigration prevents drift to local monodominance. The models differ in the relationship between the local abundances and the birth and death rates, in the relative abundances in the metacommunity, and in whether the dynamics are syncronous or sequential in time.

### Local model HL

Our local community model “HL” resembles one used by Haegeman and Loreau [33]. It can be thought of as a multispecies stochastic Lotka-Volterra competition model with immigration, where a single parameter controls the relative strength of inter-and intra-specific interactions. Our model differs from that of Haegeman and Loreau [33] in that each death event is immediately followed by a single birth event so that the local community size remains constant. We consider a local community consisting of *J* individuals, each of which has a species identity which is an integer between 1 and *S*_*T*_. Mortality is affected by inter- and intra-specific density dependent mortality, so that the probability that the next individual that dies has species identiy *i* is proportional to

$$\begin{array}{ccc} M_{i}\left(\left\{n_{j}\right\}\right) & = & \left(\frac{n_{i}}{J}+\left(1-\gamma \right)\frac{\sum_{j\neq i}n_{j}}{J}\right)\frac{n_{i}}{J}\\ & = & \left(\left(1-\gamma \right)+\gamma \frac{n_{i}}{J}\right)\frac{n_{i}}{J} \end{array}$$ (1)

where *n*_*i*_ is the number of individuals of species *i* in the community, so that $J=\sum_{j=1}^{S_{T}}n_{j}$. In the neutral case, *γ* = 0, the mortality rate is the same for all individuals irrespective of species, but when *γ* > 0 per capita mortality is greater for more abundant species.

The dynamics proceed by choosing the individual that dies next, so that the probability that the dead individual has species identity *i* is proportional to *M*_*i*_. A recruit is then chosen to be of species *i* with probability

$$\begin{array}{c} F_{i}\left(\left\{n_{j}\right\}\right)=\left(1-m\right)\frac{n_{i}}{J}+mP_{i}, \end{array}$$ (2)

where *P*_*i*_ is the relative frequency of species *i* in the metacommunity. One time step consists of *J* of these elemental update steps.

If *γ* = 0, the mortality rate is independent of species identity, so species interactions are neutral; when 0 < *γ* ≤ 1, niche differentiation tends to promote species coexistence [33]. The model is ill-defined when *γ* > 1, since that would lead to *M*_*i*_ < 0.

At first sight, it might appear that a more general model could be obtained by using the functional forms in Haegeman and Loreau [33], which allow for density independent as well as density dependent mortality. The rates in Equation (13) of that paper correspond in our notation to

$$\begin{array}{ccc} M_{i} & = & r_{-}n_{i}+\left(r_{+}-r_{-}\right)n_{i}\frac{\alpha J+\left(1-\alpha \right)n_{i}}{K^{\prime }} \end{array}$$ (3)

$$\begin{array}{ccc} F_{i} & = & r_{+}n_{i}+\mu S_{T}P_{i}, \end{array}$$ (4)

where both *M*_*i*_ and *F*_*i*_ are now rates rather than probabilities (no longer normalised so that they sum to unity) and we have introduced the factor *S*_*T*_ *P*_*i*_ to allow the immigration rate to differ between species. Here, *r*_+_ and *r*_−_ denote respectively the rates of density-independent birth and mortality, *K* ^′^ plays the role of a carrying capacity, and *α* tunes how neutral the interactions are are (neutral when 1; non-neutral competition for 0 < *α* < 1; mutualistic for *α* < 0). A little algebra shows that Equations (3) and (4) are equivalent to Equations (1) and (2) (up to overall prefactors that do not affect the sequence of processes in the simulation) with the choice of parameters

$$\begin{array}{ccc} \gamma & = & \frac{1}{1+\frac{r_{-}K^{\prime }+\left(r_{+}-r_{-}\right)J\alpha }{\left(r_{+}-r_{-}\right)J\left(1-\alpha \right)}}\\ m & = & \frac{1}{1+\frac{r_{+}J}{\mu S_{T}}}. \end{array}$$

The values of these parameters is within the range for which our model is defined (0 < *m* ≤ 1, 0 ≤ *γ* ≤ 1) provided *r*_+_ > *r*_−_ (which is assumed to be the case by Haegeman and Loreau [33] in order for there to be a non-trivial equilibrium) and $-\frac{r_{-}K^{\prime }}{(r_{+}-r_{-})J}\leq \alpha \leq 1$. Therefore, our model defined by Equations (1) and (2) captures the apparently more general density dependence defined in Equations (3) and (4), except for the case of strongly mutualistic interactions.

### Local model PC

Our second model of non-neutral species interactions is a derivative of that of Pigolotti and Cencini [42]. As in model HL, we assume that interspecific interactions are weaker among heterospecifics than among conspecifics, but in model PC we assume a Ricker-like functional form that acts on fecundity so that the number of local propagules of species *i* ∈ {1, …, *S*_*T*_} is ∝ *n*_*i*_ exp(−*an*_*i*_ − *b*∑_*i* ≠ *j*_ *n*_*j*_), where *n*_*k*_ is the local abundance of species *k* and *a* and *b*(< *a*) are constants. The fraction of local propagules that are of species *i* is then

$$\begin{array}{ccc} L_{i}^{PC} & = & \frac{n_{i}e^{-an_{i}-b\sum_{j\neq i}n_{j}}}{\sum_{k=1}^{S_{T}}n_{k}e^{-an_{k}-b\sum_{j\neq k}n_{j}}}\\ & = & \frac{n_{i}e^{-cn_{i}/J}}{\sum_{k=1}^{S_{T}}n_{k}e^{-cn_{k}/J}}, \end{array}$$

where *c* = *J*(*a* − *b*). If the interactions are sensitive to the average local density of the different species, i.e. all species are spread throughout the community, then for the same pool of species we expect *c* to be independent of *J*. Spatial effects could lead to a focal species only being sensitive to the dynamics of nearby species, in which case the effective value of *c* would depend on *J*, although this can only be modelled correctly using a spatially explicit model.

Because a fraction *m* of recruits are immigrants from the metacommunity, the probability that a new recruit is of species *i* is

$$\begin{array}{c} R_{i}=mP_{i}+\left(1-m\right)L_{i}^{PC}. \end{array}$$ (5)

In this model, we assume that the generations are discrete and non-overlapping: at each timestep, we compute the *R*_*i*_ from the current configuration, and generate a new configuration using

$$\begin{array}{c} \left\{n_{i}\right\}∼\text{multinomial}\left(J,\left\{R_{i}\right\}\right). \end{array}$$

We do this for the sake of computational efficiency: using a multinomial pseudo random number generator when the local community size is of the order of *J* ∼ 10000 a full system update of this synchronous model is orders of magnitude quicker than for the sequential update model. Sequential updating (as in model HL above) is a more faithful biological description of triopical forest dynamics, but it is known that in the neutral limit the sequential model (Moran process) and the synchronous model (Wright-Fisher process) give indistinguishable equilibrium statistics for the large community sizes we are interested in. The syncronous model is therefore well suited to our goal of exploring the detectability of departures from neutrality. The only circumstances where the synchronous model behaves qualitatively differently from the sequential one is where the Ricker-like dynamics tend to lead to limit cycles or chaos, but that does not affect the results in this paper because we always choose *c* < 2*S*_*T*_ (see Metacommunity model EVEN below).

### Local model IF

Interspecific interactions, as implemented in models HL and PC, could be interpreted as representing environmental variability within the community: each species has its own preferred microhabitat, and as a result competes less with heterospecifics (which occupy neighbouring but different microhabitats) than with conspecifics. By contrast, model IF could be interpreted as considering environmental variability between communities. In any given local community, each (out of a total number *S*_*T*_) species has a different intrinsic fitness *f*_*i*_, different from the other species and different from its intrinsic fitness in other commuities. Our model introduces intrinsic fitness differences in a similar way to Chesson and Warner [58], though our model is otherwise different because our fitness differences do not fluctuate in time and our dynamics are stochastic rather than deterministic.

For each realisation, we generate the *f*_*i*_ from a Gamma distribution with mean shape factor 1/*k*, so that *k* ^1/2^ is the coefficient of variation among the fitnesses, and all the fitnesses are equal when *k* → 0. The fraction of local propagules that are of species *i* is then

$$\begin{array}{c} L_{i}^{IF}=\frac{f_{i}n_{i}}{\sum_{k=1}^{S_{T}}f_{k}n_{k}}, \end{array}$$

and the probability that a recruit is of species *i* is

$$\begin{array}{c} R_{i}=mP_{i}+\left(1-m\right)L_{i}^{IF}. \end{array}$$

As was the case for model PC, for computational efficiency we assume discrete generations, and simulate the model using a multinomial pseudorandom number generator with probability vector {*R*_*i*_}.

### Metacommunity model LOGS

We assume that the relative immigration rates of different species reflect their abundance in a wider metacommunity. Following the SNM, in model LOGS we assume that this abundance distribution follows a Fisher logseries with diversity parameter *θ*. This is often a good description of empirical data, and can arise from several models of community dynamics including Hubbell’s neutral model [31, 59]. Note that the metacommunity represents the pool from which immigrants can be drawn, which could comprise many disparate communities. Therefore, the metacommunity does not necessarily correspond to the large-*J* limit of a single local community model. This means that one reasonable scenario is that the metacommunity follows a canonical form form due to averaging over very large scales [35], even when the local dynamics are non-neutral.

In metacommunity model LOGS we use the distribution introduced by Ewens [60] to give the number *f*_*M*_ of species in the metacommunity with relative abundance *x* within the interval (*x*; *x* + *dx*).

$$\begin{array}{c} f_{M}\left(x\right)dx=\frac{\theta }{x}{\left(1-x\right)}^{\theta -1}dx. \end{array}$$ (6)

This distribution is a continuum form of Fisher’s log-series that is appropriate when sampling from an effectively infinite metacommunity [14]. In practice, we use this distribution to simulate from a very large metacommunity containing *S*_*T*_ species; our full sampling algorithm is described in S1 Text. The results in this paper are for *S*_*T*_ = 2000, which is large enough to be effectively infinite (i.e. a choosing a larger *S*_*T*_ did not have a perceptible effect on community statistics or the power of the test of neutrality, but did increase the duration of the simulation).

### Metacommunity model EVEN

As explained above, the logseries is a reasonable candidate metacommuity model even when local community dynamics are non-neutral. However, it is also the metacommunity model in the SNM, so we want to consider the possibility that non-neutral processes are visible in the metacommunity as well. In our EVEN metacommunity model, there are *S*_*T*_ species with equal relative abundance, $P_{i}=\frac{1}{S_{T}}$. This distribution has been used in previous modeling studies of neutral and non-neutral community dynamics [33, 54, 61, 62], though it has little empirical support. It is appropriate to use this distribution in our study because, as we shall show, it represents a metacommunity limit of our non-neutral local community models.

The HL model is a form of stochastic multi-species Lotka-Volterra model, so its large-*J* limit is described by differential equations. When 0 ≤ *α* < 1, this has a stable equilibrium with $\frac{n_{i}}{J}=\frac{1}{S_{T}}$ for all *i*.

The PC model is a form of Ricker map. When *J* is large, the multinomial distribution becomes sharply peaked around its mean value, so from Equation (5) (for vanishing *m*) the dynamics of $r_{i}=\frac{n_{i}}{J}$ follows

$$\begin{array}{c} r_{i}\left(t+1\right)=\frac{r_{i}\left(t\right)e^{-cr_{i}\left(t\right)}}{\sum_{k}r_{k}\left(t\right)e^{-cr_{k}\left(t\right)}}. \end{array}$$

A standard stability analysis shows that the equilibrium $r_{i}=\frac{1}{S_{T}}$ is stable provided *cS*_*T*_ < 2; for higher values of *c* the community displays limit cycles or chaos.

In model IF, the community tends to be dominated by the species that has the highest fitness. However, the metacommunity represents an aggregate of many independent local communities, and we expect different species to dominate in different communities. The model assigns fitnesses independently at random to the different species, so we expect each species to have the same overall relative abundance $\frac{1}{S_{T}}$ in the metacommunity.

Therefore, the EVEN metacommunity model represents one metacommunity limit of our local community models. Other metacommunity models could be obtained by taking the limit in different ways. For instance, if parameter *c* in model PC depends on *J* and approaches zero sufficiently rapidly in the limit *J* → ∞, then the metacommunity limit would be neutral and follow the same Ewens distribution as model LOGS. If the fitnesses in model IF were not i.i.d. random variables, but rather different species had different mean fitnesses, then the metacommunity would have a different, uneven distribution. While there is an infinite variety of possible metacommunity distributions, the EVEN metacommunity represents the most contrasting distribution to the logseries, in the sense that it has the maximum Shannon diversity index for a given species richness while the logseries is a very uneven distribution. It also has the advantage of being characterised by a single parameter (*S*_*T*_), whereas other commonly-used distributions (e.g. the lognormal) generally require two parameters.

### Testing the neutral null model

In order to quantify whether a particular data set is consistent with neutral theory, we adopt a maximum likelihood approach together with a parametric bootstrap as used by Walker and Cyr [45] and Rosindell and Etienne [63]. To calculate the *p*-value of our test, we compare the value of a test statistic for the test data set with values of the test statistic for data sets generated by the null model. We choose the maximized likelihood of the neutral model as our test statistic. The likelihood *L*(*X*∣*m, θ*) that the neutral model would generate a data set *X*, for parameters (*m, θ*) is computed using the exact formula derived by Etienne [26]. We use code based on Tetame (http://chave.ups-tlse.fr/projects/tetame.htm), an efficient implementation in C++ of Etienne’s formula that was developed by Jabot et al. [64]. We have ported the Tetame code to C, and adapted it so it can be loaded as a dynamic library in R using the .C() function.

The hypothesis test consists of the following steps:

1. For a test data set *X*_*T*_, we find the maximum likelihood parameter estimates (*m*_*MT*_, *θ*_*MT*_), i.e. the set of parameters for which *L*(*X*_*T*_∣*m, θ*) takes its largest value, *L*(*X*_*T*_∣*m*_*MT*_, *θ*_*MT*_), the maximum likelihood estimate.
2. Generate a large number *u* (in this study, *u* = 1000) of sample data sets from a neutral model with parameters (*m*_*MT*_, *θ*_*MT*_), using an Urn algorithm [14, 26].
3. For the *i*’th sample neutral data set, compute the corresponding maximum likelihood estimate parameter set $\left(m_{MN}^{i},\theta_{MN}^{i}\right)$ and maximum likelihood $L(X_{N}^{i}|m_{MN}^{i},\theta_{MN}^{i})$ using the same procedure as used for *X*_*T*_.
4. The *p*-value for the test is the fraction of neutral data sets whose maximum likelihood is lower than the maximum likelihood for the test data set, i.e. *p* is estimated by:
  $$\begin{array}{c} p=\frac{1}{u}\sum_{i=1}^{u}\xi \left(L\left(X_{N}^{i}|m_{MN}^{i},\theta_{MN}^{i}\right)-L\left(X_{T}|m_{MT},\theta_{MT}\right)\right), \end{array}$$ (7)
  where *ξ* is the step function (*ξ* is equal to 1 if its argument is positive and 0 otherwise).
5. The neutral model is rejected if the *p*-value is less than the chosen threshold for statistical significance, which we take to be 0.05.

### Statistical power calculation for fixed non-neutral model parameters

The power of a statistical test is defined as the probability that the null hypothesis is rejected when it is indeed false. The statistical power can only be quantified by specifying an explicit model to represent the alternative hypothesis. The power can be computed by simulating many data sets from the alternative model, and performing a test of the null hypothesis on each data set, as explained above. The power is the fraction of cases for which the null hypothesis is rejected. A Type II error is defined as the failure to reject the null hypothesis when the alternative hypothesis is true, so the power is equal to 1 − *β*, where *β* is the probability of Type II errors.

The power of a test will depend on the magnitude of the deviation from the null hypothesis—the so-called effect size—and on the quality of the data at hand, typically, sample size. When coupled to the LOGS metacommunity, our models are equivalent to the SNM in the limit where the non-neutral parameter (*γ* for model HL; *c* for model PC; *k* for model IF) is zero, so *γ, c*, or *k* is our effect size for these models. When coupled to the EVEN metacommunity, our models are never strictly equivalent to the SNM so the effect size cannot be defined. In general, the power of the test will also depend on other model parameters, so we need to perform power calculations for a wide range of potentially interesting parameter values. To calculate the power of tests of neutrality for a non-neutral model with a particular set of model parameters *Y*_*T*_, we use the following procedure:

1. Generate a large number (in this study, at least 100 and usually more than 400) of equilibrium data sets from the non-neutral model with parameters *Y*_*T*_, by simulating the model for a sufficient number of time steps. The number of time steps was chosen to be at least ten times the number of timesteps such that the species richness and Shannon diversity index in the local community appeared to have reached their equilibrium values; this number depends on the model parameters (e.g. fewer time steps are needed when *m* is close to 1);
2. For each data set, perform a test of the neutral null model using the parametric bootstrap method described above.
3. The power of the test is the fraction of non-neutral data sets for which the test was significant (i.e. the neutral null model was rejected).

Because each non-neutral and neutral data set is statistically independent, the power is a binomial proportional random variable. Where shown, confidence intervals are 95% Jeffreys intervals [65].

### Power calculations for particular experiments

Our focus in this paper is on the detectability of non-neutral processes neutral in empirical situations. The power of the test is a property of the ensemble of data sets that could be produced if the data were generated by a non-neutral alternative model. This ensemble of data sets depends on the model parameters which are chosen, but for a particular data set we do not necessarily know the appropriate parameters to use in the alternative model. Here, we choose parameter sets such that the model best describes a set of summary statistics of empirical data sets, specifically: species richness and Shannon index. Once the strength of the non-neutral process (*α, c*, or *k* as appropriate) and the local community size *J* are chosen, the model is characterised by two further parameters: the immigration rate *m* and the diversity (*θ* for model LOGS or *S*_*T*_ for model EVEN) of the metacommunity. There will therefore be a discrete set of parameter values where the mean species richness and mean Shannon index of samples from the model match the empirical data sets; in most cases we found only one such parameter set, though in some cases there were two and in others there were none because the model produced a Shannon index that was always higher than, or always lower than, the empirical data.

We performed this procedure, for a set of candidate values of the non-neutral parameter, to generate parameter sets resembling three tropical forest data sets belonging to the CTFS network to which neutral theory has successfully been fitted in the past [46]: Barro Colorado Island, Pasoh Forest Reserve, and Lambir Hills National Park [37–39]. For each forest, and separately for each survey year, we tested the null model that the data were generated by the SNM using the parametric bootstrap method described above. In each case we found *p* > 0.05, showing that the data were statistically consistent with SNM. The mean total community size, species richness, and Shannon index for these sites, averaged over the census years available at http://www.ctfs.si.edu, are given in Table 5. These sites were selected because they had higher Shannon index than a logseries distribution with the same size and richness, so we expected that a model with non-neutral interspecific interactions or an EVEN metacommunity would describe the data better than the SNM.

**Table 5:** Summary statistics for the three tropical forest data sets to which our non-neutral models were fitted.

Data set	*J*	*S*	Shannon index
Barro Colorado Island (1982–2005)	21058	232	4.275
Pasoh Forest Reserve (1987–2000)	27955	672	5.657
Lambir Hills National Park (1992–1997)	32918	1007	5.928

Volkov et al. [46] have compared the SNM to three other CTFS forest sites, but the Shannon index in these sites is lower than (Korup National Park and Yasuni National Park) or almost equal to (Sinharaja World Heritage Site) that of a logseries, so we expected it to be more difficult to find suitable model parameters. We also found that the Yasuni National Park data were not consistent with SNM (*p* = 0.001 for 1996 and *p* = 0.014 for 2003), though we found *p* > 0.05 for all Korup and Sinharaja surveys.

## Supporting Information

S1 Text Sampling from an infinite metacommunity. (PDF)
