# Supplementary material for: When Can Species Abundance Data Reveal Non-neutrality?
Source: PLoS Comput Biol. 2015 Mar 20;11(3):e1004134. doi: 10.1371/journal.pcbi.1004134 (PMC4368519; doi:10.1371/journal.pcbi.1004134)
Supplement: S1 Text — (PDF) [file pcbi.1004134.s001.pdf]

S1 Sampling from an infinite metacommunity

To perform simulations of the LOGS metacommunity, we need an algorithm to obtain the relative abundance of species i in the metacommunity, P_i. There is a known algorithm to sample individuals from an infinite metacommunity but generating a large community would be a slow way to obtain P_i. The starting point of the algorithm is equation 6 which describes the metacommunity at the speciation-extinction equilibrium [14,48,60]:

$$f_M(x)dx = \frac{\theta}{x}(1-x)^{\theta-1}dx. \tag{S.1}$$

$f_M(x)dx$ is the probability of extracting a species of relative abundance in the interval $(x; x+dx)$. θ is the fundamental biodiversity parameter obtained by combining the metacommunity size and the speciation rate [31].

To obtain P_i we first solve the equations :

$$\int_{y_n}^{1} f_M(x)dx = n \tag{S.2}$$

for $n \geq 1$. We keep the values of y_n for n in the interval $[1 : S_T]$ which is the number of species we consider in the metacommunity. S_T has to be introduced as otherwise we would have an infinite array that we could not store. y_{S_T} is the inverse population size in the metacommunity $\frac{1}{J}$. S_T can be taken as large as desired and equation S.2 can be solved very easily for large values of n thanks to an asymptotic approximation for small x. A large enough S_T is effectively equivalent to an infinite metacommunity for a finite simulation, because rare species are exponentially suppressed.

From equation 6 the probability of sampling an individual from a species of relative abundance in the interval $(x; x+dx)$ is [60] given by $p(x)dx$ where :

$$p(x)dx = xf_M(x)dx = \theta(1-x)^{\theta-1}dx. \tag{S.3}$$

The second step is to integrate equation S.3 in the interval $[y_{n+1}; y_n]$ to obtain the probability of sampling an individual in the previously defined integer species group defined in step (1). Doing this we obtain:

$$P_i = \int_{y_{n+1}}^{y_n} \theta(1-x)^{\theta-1}dx = (1-y_{n+1})^{\theta} - (1-y_n)^{\theta}. \tag{S.4}$$

This last expression is already normalized if the number of species is infinite but in the case it is finite the total $\sum_i P_i = P_{tot}$ is less than 1 (but close to it) we normalize it by dividing by P_{tot}. The array of P_i obtained by combining the previous two steps is what we use throughout this work. Notice that the last expression can also be regarded as the expected relative frequency of a species in a specified integer species group, so the P_i sequence corresponds to the expected rank abundance curve of an infinite metacommunity. If we sampled from the species pool according to the full infinite set of P_i, we would have an algorithm which is equivalent to the one developed by Ewens [31,48,60]. In practice, our algorithm is an approximation because (for reasons of computational efficiency) we truncate at a finite S_T.
